# Cognitive Processing Speed in Older Adults: Relationship with White Matter Integrity

**DOI:** 10.1371/journal.pone.0050425

**Published:** 2012-11-21

**Authors:** Geoffrey A. Kerchner, Caroline A. Racine, Sandra Hale, Reva Wilheim, Victor Laluz, Bruce L. Miller, Joel H. Kramer

**Affiliations:** 1 Stanford Center for Memory Disorders, Department of Neurology and Neurological Sciences, Stanford University School of Medicine, Stanford, California, United States of America; 2 Departments of Neurological Surgery and Radiation Oncology, University of California, San Francisco, California, United States of America; 3 Cognitive Development Laboratory, Department of Psychology, Washington University in St. Louis, St. Louis, Missouri, United States of America; 4 Memory and Aging Center, Department of Neurology, University of California, San Francisco, California, United States of America; University Of Cambridge, United Kingdom

## Abstract

Cognitive processing slows with age. We sought to determine the importance of white matter integrity, assessed by diffusion tensor imaging (DTI), at influencing cognitive processing speed among normal older adults, assessed using a novel battery of computerized, non-verbal, choice reaction time tasks. We studied 131 cognitively normal adults aged 55–87 using a cross-sectional design. Each participant underwent our test battery, as well as MRI with DTI. We carried out cross-subject comparisons using tract-based spatial statistics. As expected, reaction time slowed significantly with age. In diffuse areas of frontal and parietal white matter, especially the anterior corpus callosum, fractional anisotropy values correlated negatively with reaction time. The genu and body of the corpus callosum, superior longitudinal fasciculus, and inferior fronto-occipital fasciculus were among the areas most involved. This relationship was not explained by gray or white matter atrophy or by white matter lesion volume. In a statistical mediation analysis, loss of white matter integrity mediated the relationship between age and cognitive processing speed.

## Introduction

From midlife onwards, cognitive processing speed declines with age [1], [2]. This slowing occurs in otherwise healthy, normal adults who show no sign of a neurodegenerative disease, and it leads to loss of function and other morbidity. For example, slowed processing speed is the most important predictor of driving cessation in the elderly [3].

The cause of age-related cognitive slowing remains unclear. One hypothesis is that it relates to another age-related phenomenon, the loss of cerebral white matter integrity as detected by diffusion tensor imaging (DTI). Multiple groups have demonstrated correlations between DTI metrics and age [4], [5], [6], [7], [8], [9], [10], [11], [12], [13], [14], [15], [16], [17]. The exact nature of age-associated white matter change is probably multifactorial, including some combination of microscopic disruption of myelin or of axons themselves, gross changes in white matter volume, or the accumulation of lesions that are visible by T2-weighted MRI and are normally attributed to chronic ischemia.

DTI metrics in many fiber tracts of the brain correlate with simple response time on basic perceptual and motor tasks [12], [16], [18], [19] and on timed performance on more cognitively challenging tests of executive function or working memory [6], [9], [20], [21], [22], [23], [24], [25], [26], [27], [28], [29]. In these prior studies, the term “processing speed” has been used in relation to performance on a broad array of tasks, relying variably upon motor skills and various cognitive skills.

To extract the purest possible measure of cognitive processing speed, we designed a series of computerized, binary choice reaction time behavioral tests with the following considerations: First, our tasks relied upon nonverbal visuospatial cues, because prior work revealed an age-dependent slowing on such tasks more reliably than on verbal tasks [2], [30], [31]. Second, time spent on a task includes separate cognitive and motor components; tasks that require either very limited cognitive processing (e.g., press the button when the target appears) or a complex motor output (e.g., a pegboard test or handwritten responses on the Digit Symbol subtest of the Wechsler Adult Intelligence Scale) may be biased by motor speed. Our binary button-press tasks were designed to be motorically simple while simultaneously varying cognitive difficulty, so that true cognitive processing occupied the bulk of the response latency interval. Third, an extensive review of the literature revealed that the most valid measure of processing speed is obtained by administering multiple tasks rather than relying on reaction times from a single task [32].

We administered these tests to healthy older adults, normalized their scores to a group of young controls, and investigated the relationships between age, processing speed, and cerebral white matter integrity. Using tract-based spatial statistics, an unbiased data-driven method for DTI group analysis [33], we investigated the relationship between processing speed and various diffusion indices: fractional anisotropy (FA; an index ranging from 0, indicating isotropic diffusion or equal movement in all directions, to 1, indicating diffusion along a single vector), mean diffusivity (MD; the apparent diffusion coefficient, a directionless measure of water diffusion), radial diffusivity (DR; the extent of diffusion orthogonal to the principal diffusion direction), and axial diffusivity (DA; the extent of diffusion along the principal direction) [34]. We also compared processing speed to white matter lesion load and to the extent of white and gray matter atrophy.

## Subjects and Methods

### Subjects

We prospectively recruited 174 healthy adults aged 55 and older from existing research cohorts at the UCSF Memory and Aging Center and from the community. Each potential subject underwent a complete history, neurological examination, a functional assessment, and an hour-long neuropsychological screening battery, followed by a consensus conference for the determination of diagnosis and suitability for the study. Subjects were excluded on the basis of a Clinical Dementia Rating score [35] greater than zero, symptoms of cognitive impairment (endorsed by the patient or a well-acquainted informant), or findings during examination that were concerning for incipient cognitive decline. Additional exclusion criteria included any contraindication to MRI; any sensory or motor disability that may have prevented participation or cooperation with the study protocol; any history of brain tumor, Parkinson’s disease, or multiple sclerosis; any active substance abuse; or any evidence or history during the previous 2 years of epilepsy, focal brain lesion, head injury with loss of consciousness or immediate confusion, cancer (except for non-melanoma skin cancer), steroid use, or major psychiatric disorder, including psychosis, major depression, bipolar disease, or alcohol or substance abuse. Table 1 details the disposition of recruited subjects, and the characteristics of the 131 subjects ultimately included in this study.

**Table 1:** Subjects.

**Number recruited**	174
**Failed screening** *	29
**Withdrew**	2
**Structural defect** **	2
**Incomplete data**	10
**Number included**	131
**Age (range)**	69.6±5.9 (55–87)
**Male:Female**	53∶78
**Handedness (right:other** *** **)**	113∶18
**MMSE**	29.3±0.8
**Education**	17.5±2.1 years

* See exclusion criteria in Subjects and Methods.

** In these subjects, MRI revealed an unexpected brain lesion.

*** Other includes left-handed (11), ambidextrous (3), and not known (4).

MMSE, Mini Mental State Exam.

### Ethics Statement

Each participant provided written, informed consent. The consent form and this study protocol were approved by the UCSF Committee on Human Research.

### Processing Speed Testing

**Figure 1:**
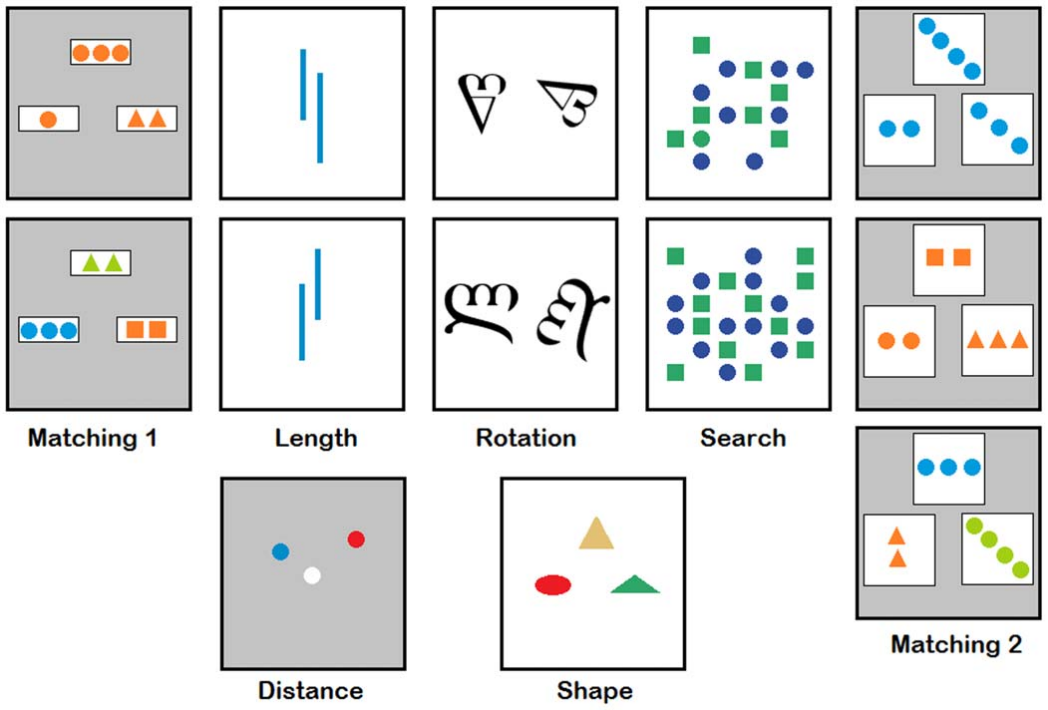
Computerized processing speed tasks. Screenshots are illustrated for the seven visuospatial choice reaction time tests described in detail in the text (Subjects and Methods).

We administered, in a fixed order, seven visuospatial tasks [30], [36] that each included 1–4 conditions for a total of 14 task conditions, as described below and in Figure 1 and Table 2. For each older subject on each task condition, a scaled response latency z-score was calculated by comparing the mean reaction time to the average and standard deviation of the mean reaction times on that task condition among a sample of young adult controls (n = 40; 16 males; age 24±3.1 years; education 16±1.6 years). Averaging these 14 z-scores for each older subject yielded a composite z-score. This approach – comparing mean reaction times between older subjects and younger controls to infer processing speed – has previously been validated [37], [38]. We also performed a principal components analysis over the whole data set of reaction times on each subtest, and found a latent factor that correlated very closely with our composite z-score (r = 0.94; p<0.001).

**Table 2:** Processing speed tasks and subtests.

Tasks*	Subtest*	Accuracy (%)	Mean response latency (sec)
**Abstract Matching 1:**	–	91±6	1.98±0.86
**Distance Judgment:**	–	99±2	0.87±0.18
**Length Judgment:**	20%*	99±3	0.68±0.14
	10%	97±5	0.83±0.23
**Mental Rotation:**	60°	82±33	2.57±0.84
	120°	78±32	3.03±0.98
**Visual Search:**	16, no target	99±3	1.87±0.59
	16, with target	91±9	1.15±0.22
	24, no target	99±3	2.35±0.80
	24, with target	90±9	1.33±0.29
**Shape Judgment:**	–	99±3	1.16±0.28
**Abstract Matching 2:**	3-dimension	94±10	2.90±0.92
	2-dimension	94±11	3.77±1.40
	1-dimension	94±12	3.46±1.22

* See Subjects and Methods and Figure 1 for explanations of all tests and subtests. Accuracy reflects the percentage of correct responses during the experimental trials (not the practice trials). Similarly, mean response latency is for all responses during the experimental trials.

Experiments were programmed on a PC using Eprime software (Psychology Software Tools, Inc., Sharpsburg, PA; http://www.pstnet.com/eprime.cfm), and participants were asked to make visuospatial judgments about stimuli presented on the display. Standard instructions were for subjects to respond as quickly and accurately as possible. Subjects were asked to use the index and middle finger of their right hand to press the key on a keyboard that corresponded with their answer choice. All tasks required a binary decision – yes/no or left/right. Each task began with 5–10 practice trials; if accuracy on the practice trials was below 70%, subjects carried out an additional round of practice trials before proceeding. The first two experimental trials in each task condition were ‘buffer’ trials that were not included in the analyses. The median response latency on the following 20 experimental trials of each task condition was recorded for analysis. Table 2 lists the average accuracies and response latencies across tasks.

#### Abstract matching 1

Participants were presented with three arrays of shapes: two choice arrays – a match and a foil – positioned below a third, sample array. They had to decide which of the two choice arrays was most similar to the sample array, then press the corresponding button (left or right). The arrays varied on three dimensions: shape, number, and color. The match always had one more dimension in common with the sample array relative to the foil.

#### Distance judgment

Participants were shown a white circle located approximately in the center of the display, and two additional circles (blue and red) located on the left and right hand-sides of the screen at least one inch away from the center point of an imaginary vertical line that bisects the white center dot. The participant had to judge which of the two additional circles was closest to the white central circle, and push the corresponding key (left or right). The ratio of the distances of the further to the closer dot ranged from 4∶1 (easy) to 6∶5 (hard).

#### Length judgment

Participants were shown two vertical parallel lines of different lengths, then asked to determine which line was longer and press the corresponding button (left or right). The two lines were displayed such that neither the tops nor bottoms were aligned, thereby requiring participants to make a judgment about actual line length rather than a simple height judgment. For this task, two conditions were equally presented: (1) lines differed in length by 20%, and (2) lines differed in length by 10%.

#### Mental rotation

Participants were shown a letter from the Georgian Cyrillic alphabet that appeared twice on the screen, on the left in its proper (upright) position and on the right in a rotated position; in half of the trials, the right, rotated letter was a mirror-image of the letter on the left so that it did not match the left letter. Participants had to determine whether, after rotation, the two letters were identical (as opposed to being mirror images), and then choose the corresponding key (yes or no). For this task, two conditions were equally presented: (1) the letter on the right would need to be rotated 60° to be upright, and (2) the letter on the right would need to be rotated 120° to be upright.

#### Visual search

Participants were shown arrays of blue circles and green squares. Subjects had to determine whether a target (a green circle) appeared on the screen and then press the corresponding button (yes or no). Four conditions were equally presented: (1) an array of 16 objects without a target, (2) an array of 16 objects with a target, (3) an array of 24 objects without a target, and (4) an array of 24 objects with a target.

#### Shape judgment

Participants were shown a single shape on top and two additional shapes below. They had to judge which of the two lower shapes (left or right) was most similar to the shape on top and then press the corresponding button (left or right). Each shape was shown in a different color, but color was not relevant to the choice.

#### Abstract matching 2

This task was similar to Abstract Matching 1, except that four dimensions were used instead of three: shape, number, color, and orientation. For this task, three conditions were equally presented: (1) match and sample equivalent on three dimensions, (2) match and sample equivalent on two dimensions, and (3) match and sample equivalent on only one dimension.

### Episodic Memory Testing

One component of the neuropsychological screening battery (see Subjects and Methods) was the California Verbal Learning Test II (CVLT-II), a 16-item word list learning task. Delayed recall was quantified as the number of words recalled after a 20 minute delay.

### Structural Imaging Acquisition and Processing

Each subject underwent MRI on a 3T Siemens scanner, using a protocol that included a T1-weighted 3D MPRAGE sequence (TR/TE/TI 2300/3/900 ms; flip angle 9°; sagittal acquisition with FOV 256×240 mm^2^ and 1 mm thick slices; matrix 256×240 with 160 slices yielding 1 mm^3^ isotropic voxels). Total intracranial volume (TIV) was calculated using the BET and FAST tools from the FSL 4.1 software package [39] (http://www.fmrib.ox.ac.uk/fsl/) to segment the brain into gray matter, white matter, and CSF; TIV was calculated as the sum of the three tissue types. Because not all subjects had a fluid attenuation inverse recovery (FLAIR) or T2 sequence, the T1 scan was also used to derive white matter hypointensity volume, calculated from an automated subcortical segmentation routine using Freesurfer [40] (http://surfer.nmr.mgh.harvard.edu/). Gray and white matter voxel-based morphometry (VBM) were carried out using SPM8 [41] (http://www.fil.ion.ucl.ac.uk/spm/), following the standard processing stream, using the Segment routine to generate gray and white matter partial volume maps, then DARTEL for spatial normalization; default parameters were used throughout.

### DTI Acquisition, Processing, and Tract-based Spatial Statistics (TBSS)

The MRI evaluation also included a DTI sequence (TR/TE 8000/109 ms; B = 0 image and 64 directions at B = 2000 s/mm^2^; FOV 220×220 mm^2^ and 2.2 mm thick slices; matrix 100×100 with 55 slices yielding 2.2 mm^3^ isotropic voxels). Raw DTI data entered a standard processing stream using FSL 4.1. After eddy current correction with eddy_correct (using default parameters), diffusion tensors were fit with dtifit. Voxel-wise statistical analysis of the diffusion tensor data was carried out using TBSS [33], part of FSL 4.1. FA maps were brain-extracted then aligned to the default FSL template using a nonlinear registration tool (FNIRT). The resulting mean FA image was thinned to create a mean FA skeleton representing the centers of all tracts, using a threshold of 0.2. Each subject’s aligned FA map was projected onto this skeleton, and the resulting data was fed into voxel-wise cross-subject statistics.

### White Matter Tract Region-of-interest (ROI) Analysis

To identify specific white matter regions-of-interst (ROIs), we used a probabilistic tractography atlas [42], thresholded at 0.25, to parcellate each individual’s skeletonized FA map in standard MNI space. In other words, for each white matter tract ROI, we identified every voxel within the TBSS white matter skeleton that had at least a 25% chance of belonging to that tract. Onto each white matter ROI, we projected the map, derived from the output of the TBSS analysis described above, of all voxels within the overall white matter skeleton in which there was a highly significant inverse correlation between FA and response latency (p<0.01 after family-wise error correction). Each white matter ROI thus contained a mixture of “significant” and “non-significant” voxels. We divided the number of “significant” voxels in each white matter ROI by the total number of voxels within that ROI to derive a simple index of the degree to which each ROI accounted for the relationship between FA and processing speed.

### Statistics

Data are shown as mean ± standard deviation, and p<0.05 was the threshold for statistical significance. For DTI data, voxel-wise statistics were carried out using 5000 iterations of a random permutation method that employed threshold-free cluster enhancement (randomise, part of FSL) based on a general linear model design matrix that included age, gender, education, and TIV as nuisance variables except as indicated in the text. For VBM, SPM8 was used to run voxel-wise statistics using the same nuisance variables. In both cases, family-wise error correction was used to control for multiple comparisons. For nonimaging correlational and partial correlational analyses, Pearson correlation coefficients were calculated using the SPSS statistics software package (IBM, Armonk, NY; http://www.spss.com/).

For the mediation analysis, we extracted a single scalar FA value for each subject as follows: Using the voxels in which there was a highly significant relationship (p<0.01 after family-wise error correction) between age and FA as a mask, an average FA value over all of the voxels within that mask was derived for each subject. The significance of the indirect pathway was tested using 5000 iterations of a bootstrap approach in a SPSS macro [43].

## Results

### Processing Speed Declines with Age

**Figure 2:**
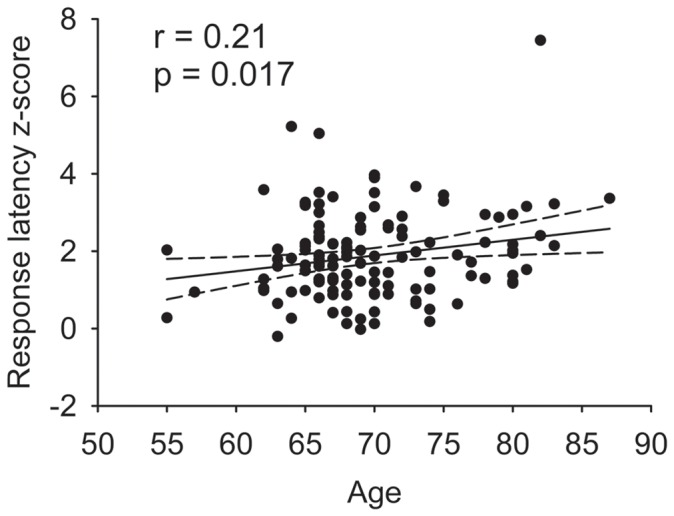
Processing speed correlates with age. A composite response latency score, calculated as a z-score relative to young normal controls, is plotted against age for the 131 subjects in this study. The line represents the linear regression, bounded by a 95% confidence interval.

Compared to the young adult controls, the older adult subjects performed on average 1.9±1.1 standard deviations more slowly on the processing speed task battery. In addition, we found that within our subjects, who ranged in age from 55 to 87 (Table 1), the composite response latency z-score correlated directly and significantly with age (Figure 2). Thus, an age-dependent slowing of cognitive processing emerged not only in comparison to young adults but also as a continuous function within this group of older adults.

### White Matter Integrity Correlates with Cognitive Processing Speed

**Figure 3:**
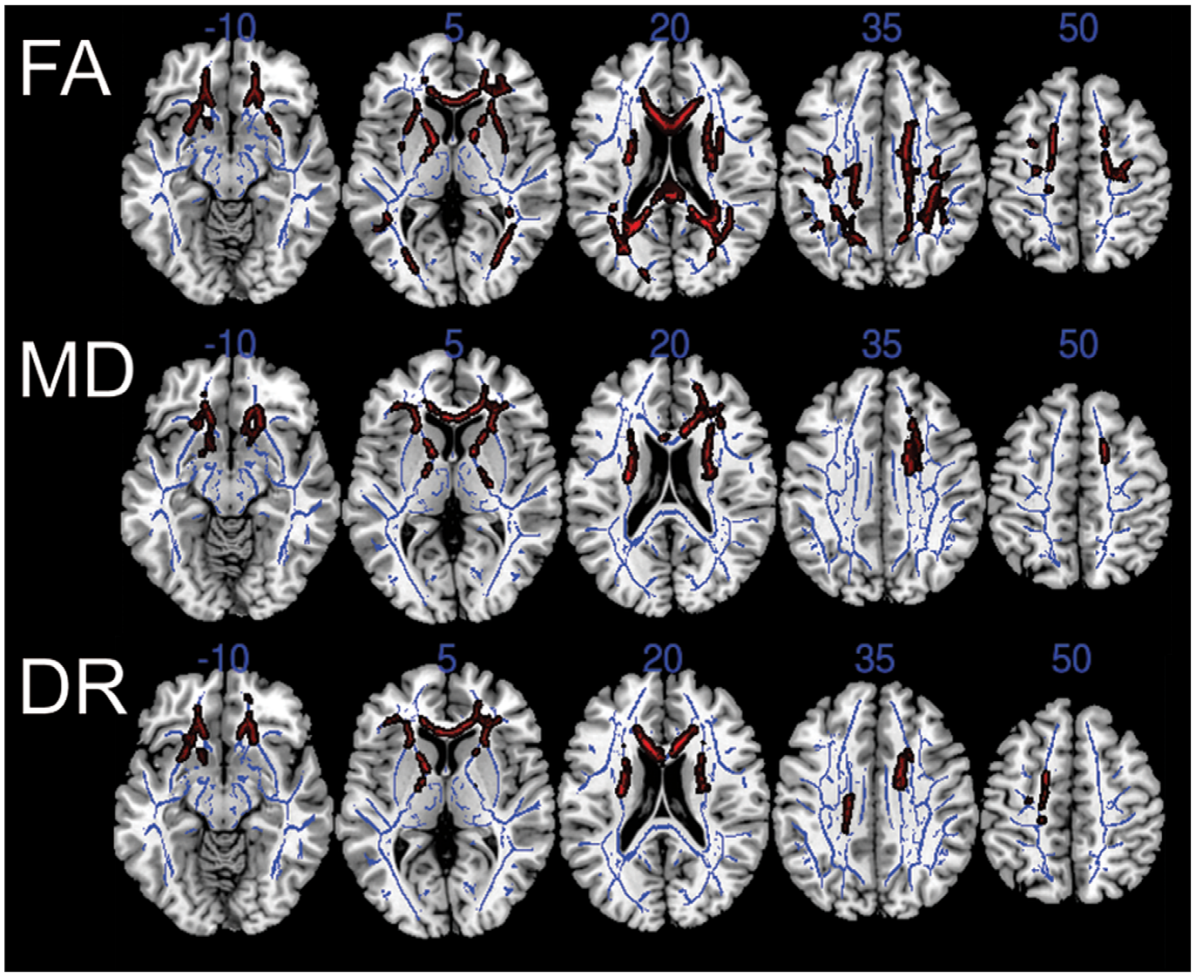
Processing speed correlates with white matter integrity. Voxel-wise regressions compared the composite scaled reaction time with various parameters of white matter integrity: fractional anisotropy (FA), mean diffusivity (MD), and radial diffusivity (DR). In red are voxels that correlated with scaled reaction time (p<0.01 after family-wise error correction); correlations were negative for FA and positive for MD and DR. These significant areas are thickened for ease of illustration. The TBSS white matter skeleton used for voxel-wise comparisons is illustrated in blue on axial images. Regression models included age, gender, education, and TIV as nuisance variables. Axial diffusivity was also tested, but was not illustrated because there was no area of significance (p>0.05). Axial slices are illustrated in anatomical (left-is-left) orientation.

We found that response latencies correlated with white matter integrity as measured by FA (negative correlation), MD (positive), and DR (positive) (Figure 3). There was no significant correlation with DA. Processing speed corresponded to white matter integrity in broad areas of the cerebral hemispheres (Figure 3). For MD and DR, the frontal lobe white matter was most involved; for FA, parietal white matter was also significantly involved (Figure 3). To ensure that the relationship observed between FA and processing speed was not driven by the three individuals with response latency z-scores >5 (see Figure 2), we performed the analysis again, leaving these three individuals out, and found a similar pattern of significant voxels. Including handedness (see Table 1) in the voxel-wise regression also did not affect these results.

**Figure 4:**
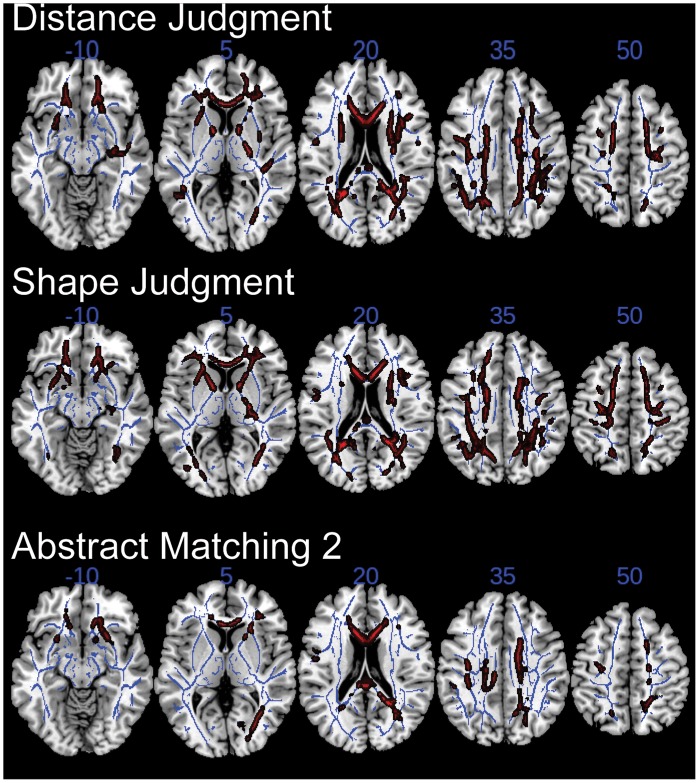
Individual processing speed tasks correlate with white matter integrity. Voxel-wise regressions compared scaled reaction time from the indicated tasks (see Subjects and Methods) with FA. In red are voxels that correlated with scaled reaction time (p<0.05 after family-wise error correction); correlations were negative. These significant areas are thickened for ease of illustration. The TBSS white matter skeleton used for voxel-wise comparisons is illustrated in blue. Regression models included age, gender, education, and TIV as nuisance variables. Results from the other four tasks are not illustrated because there was no area of significance (p>0.05). Axial slices are illustrated in anatomical (left-is-left) orientation.

We tested whether reaction times from individual tasks contributed differentially to the relationship between response latency and FA. Whereas the composite response latency z-score had a strong negative correlation with FA, such that the data illustrated in Figure 3 are thresholded at p<0.01 after family-wise error correction, data from none of the seven individual tasks yielded correlations that met this threshold. Response latencies for Distance Judgment, Shape Judgment, and Abstract Matching 2 each correlated negatively with FA at a threshold of p<0.05 after family-wise error correction (Figure 4). Results from Abstract Matching 1 revealed a trend (p<0.1), and the other tasks did not yield significant correlations. The patterns of white matter involvement were similar across the three significant tasks (Figure 4).

### White Matter ROIs

**Figure 5:**
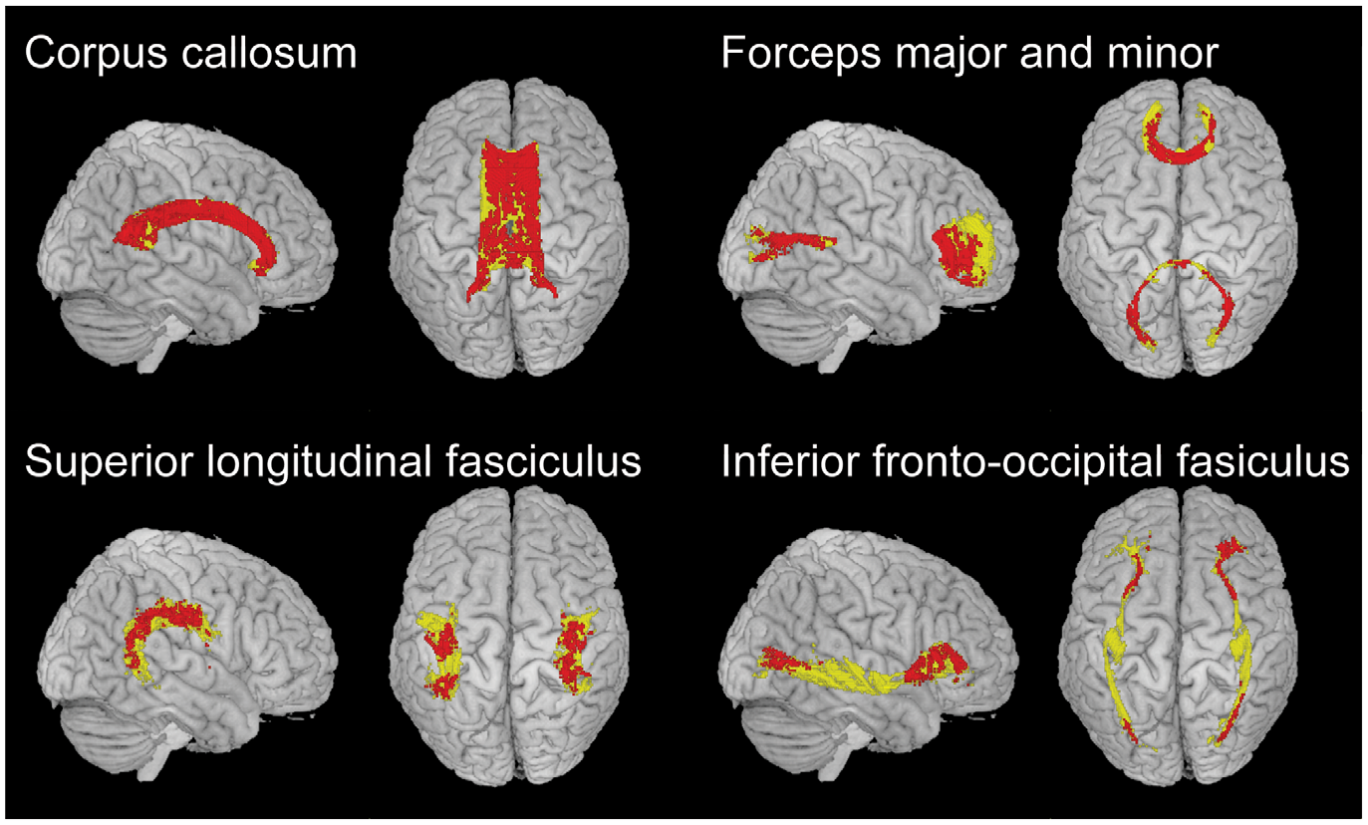
Relationship of processing speed with the integrity of individual white matter regions of interest. In yellow are voxels from the TBSS white matter skeleton contained within each indicated white matter ROI, according to a probabilistic atlas (see Subjects and Methods). Superimposed in red are the voxels within each ROI in which FA correlated inversely with scaled reaction time (p<0.01 after family-wise error correction). Forceps major and minor overlap with and contain fibers from the splenium and genu of the corpus callosum.

FA associated with processing speed most strongly in the genu and body of the corpus callosum (Table 3 and Figure 5). Other important white matter ROIs included the superior longitudinal fasciculus (SLF; including both frontoparietal and temporal portions) and the inferior fronto-occipital fasciculus (Table 3 and Figure 5). In a separate analysis using age (rather than reaction time) in a voxel-wise regression with FA, a similar pattern appeared (Table 3). However, in the SLF, processing speed but not age was a significant predictor of white matter integrity (Table 3), and in the inferior longitudinal fasciculus (ILF), the opposite situation emerged (Table 3).

**Table 3:** White matter regions of interest.

Tract		Significant Voxels (%)	
		Speed	Age
**Corpus callosum:**	GenuBodySplenium	727149	956229
**Forceps:**	MajorMinor	5538	5184
**Superior longitudinal fasciculus:**	FrontoparietalTemporal	3131	34
**Inferior fronto-occipital fasciculus**		24	46
**Anterior thalamic radiations**		20	11
**Uncinate fasciculus**		19	30
**Corticospinal tract**		8	13
**Inferior longitudinal fasciculus**		4	30
**Cingulum:**	PericallosalHippocampal	00	40

As described in more detail in Subjects and Methods, the numerical data here indicate the proportion of voxels within each white matter ROI, in which FA exhibited a significant relationship with processing speed or age (p<0.01 after family-wise error correction). ROIs are listed in decreasing order of this percentage, and some are illustrated in Figure 5. Forceps major and minor overlap with the splenium and genu of the corpus callosum (see Figure 5).

### Atrophy does not Account for the Relationship between White Matter Integrity and Processing Speed

**Figure 6:**
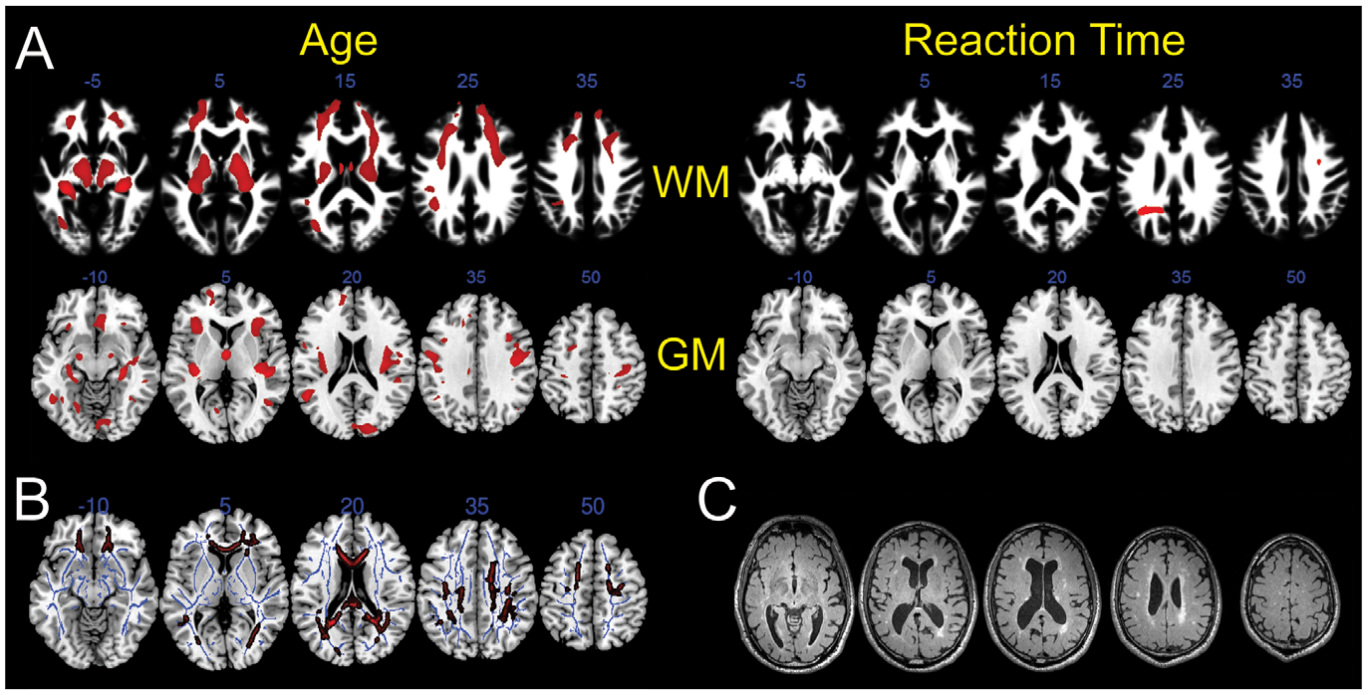
Processing speed is independent of white or gray matter atrophy. Axial image slices are illustrated in anatomical (left-is-left) orientation. (A) In red are areas of white matter (WM) or gray matter (GM) that exhibited a significant degree of volume loss in relation to age or scaled reaction time by voxel-based morphometric analysis (p<0.05 after family-wise error correction). White matter maps are superimposed on the white matter template derived from the subjects in this study, and the gray matter maps are superimposed on a standard brain template. (B) Red voxels indicate where scaled reaction time correlated inversely with FA (p<0.01 after family-wise error correction), after taking into account any contribution from white matter atrophy by entering white matter partial volume maps into the regression as a voxel-wise nuisance variable. As in Figure 3, other standard nuisance variables (age, gender, education, and TIV) were also included in the model. (C) Axial FLAIR images are illustrated for the individual in this study with the greatest extent of white matter signal change. Not all subjects in this study had a FLAIR sequence, and the T1 scan was used for determination of white matter lesion volume, as detailed in Subjects and Methods.

Not surprisingly, there was a broad negative correlation between age and both white and gray matter partial volume measurements (Figure 6A). By contrast, processing speed correlated with white matter volume in only one small area of the left parietal lobe (Figure 6A), and it did not correlate with gray matter volume in any area of the brain. Furthermore, the relationship between FA and processing speed persisted even when the subjects’ partial white matter volume maps were included as a voxel-wise nuisance variable in the regression model (Figure 6B) [44].

### White Matter Lesion Volume does not Account for the Relationship between White Matter Integrity and Processing Speed

White matter lesions, including those that are thought to result from microvascular ischemic disease, could affect processing speed or DTI parameters. Potential subjects were excluded from this study if they had a history of stroke (see Methods), and the subjects enrolled exhibited very few white matter lesions, quantified by white matter hypointensity volume on the T1 scan (see Methods). A T2-FLAIR scan from the subject with the greatest ratio of white matter lesion volume to total white matter volume is illustrated in Figure 6C. Over the whole data set, there was no significant correlation between response latency and white matter lesion volume, controlling for age and TIV (r = 0.12, p = 0.22).

### The Relationship between Age and Processing Speed is Mediated by White Matter Integrity

Using age as the independent variable, the composite response latency z-score as the dependent variable, and FA as the proposed mediator, the indirect pathway was significant at p<0.01. In this analysis, as predicted from the voxel-wise analysis (Figure 3), FA correlated inversely with response latency even when controlling for age (p = 0.0015). However, the relationship between age and reaction time was no longer significant after controlling for FA (p = 0.58). Taken together, these analyses indicate that FA mediates the relationship between age and processing speed.

As a contrast to this important role for white matter integrity in determining cognitive processing speed, FA had no bearing on verbal episodic memory, a cognitive skill that also declines with age. Delayed recall on the CVLT-II correlated inversely with age (r = –0.23, p = 0.01); however, there was no correlation between this memory score and FA (r = 0.14, p = 0.12) or processing speed (r = –0.067, p = 0.45).

## Discussion

We found that loss of white matter integrity was a significant cause of age-related cognitive slowing among otherwise cognitively normal older adults. Age-related reduction of cognitive processing speed was separate from other significant age-related processes that we observed in the same subjects, including gray and white matter atrophy, the accumulation of white matter lesions, and a decline in verbal episodic memory capacity. Thus slowing of cognitive processing speed with age emerged as a distinct phenomenon mediated by changes in DTI measures of white matter integrity.

Although we are not the first to show that FA and other DTI metrics decline with age (Table 3) [4], [5], [7], [8], [9], [10], [11], [13], [14], [15], [17] and correlate with reaction time [6], [12], [16], [18], [19], [20], [22], [24], [27], our study was large (N = 131) and methodologically rigorous in the way that cognitive processing speed was tested. Specifically, we designed tasks that minimized the contribution of motor speed to the overall response latency interval. When measuring the time required to press a button after a simple perceptual task, or when timing performance on a task that requires handwriting or other complex motor activities, motor performance has a significant potential to influence reaction time. Our tasks required cognitively complex judgments but were motorically simple. As a further refinement, we averaged the performance on multiple tasks to extract a single metric of cognitive processing speed for each participant. Finally, because our tasks were computerized, they were not examiner or site dependent, and were thus amenable to easy reproducibility. On the other hand, it is important to note that we did not perform a direct comparison between our method and other common methods of processing speed evaluation (e.g., the pegboard test or Digit Symbol test), and we cannot conclude that those approaches are invalid or would give different results. It is not possible to separate, with absolute precision, cognitive processing speed from motor ability or other cognitive attributes. For instance, our tasks still required a button press. In addition, our tasks relied heavily on visuospatial processing, and slow reaction time in some participants could conceivably have resulted from subclinical dysfunction of the visual processing stream, such as very early neurodegeneration in the right parietal lobe, although we note that there was no evidence of gray matter atrophy there or elsewhere that correlated with task performance (Figure 6A). In sum, we observed a very tight correlation between performance on our computerized tasks and FA, but it is nevertheless unlikely that white matter integrity is the sole factor accounting for the observed variance in our data.

Using an unbiased, data-driven approach with TBSS, we found that a significant relationship between DTI metrics and cognitive processing speed emerged across a broad swath of cerebral white matter, including especially the genu and body of the corpus callosum and areas of the frontal lobes (Figure 3, and Table 3). Age-related changes to these same areas have been described by others [5], [14], [15]. Our data thus support a general model in which the white matter tracts that are last to myelinate are among the earliest to deteriorate with age, a hypothesis recently tested in another TBSS study [45]. Interestingly, cognitive processing speed exhibits a similar relationship with age, improving over the first two decades of life and declining from middle age onward [1]. The convergent developmental timelines of white matter integrity and processing speed corroborate the important relationship between the two. The anatomical patterns of age-related and speed-related variability in FA were similar, except for the SLF and ILF (Table 3): Whereas many voxels within the SLF related speed to FA, the integrity of this ROI did not vary substantially with age. The significance of this finding is not evident from our study, and one direction for future work could be to investigate the specific determinants of SLF integrity among older adults. By contrast, the integrity of the ILF appeared to vary substantially with age but not with processing speed (Table 3), suggesting that our tasks were not dependent upon this particular ROI. Although individual tasks within our computerized processing speed battery contributed differentially to the relationship between response latency and FA, the overall distribution of involved white matter was not clearly distinct between tasks (Figure 4).

How changes in DTI metrics and changes in cognitive processing speed correspond to histopathological features of white matter is not known. Some have used DR as a proxy for myelin integrity, as dysmyelination might be expected to enhance water diffusion across axons [46], [47]; using similar logic, DA has been used as a proxy for axonal integrity [46], [47]. In our study, reduced cognitive processing speed was associated with increased DR but not with any change in DA, suggesting dysmyelination as a possible contributing factor. This finding is in agreement with one past study [19], but contrasts with another recent report showing that DA but not other diffusion indices correlated with processing speed, and that this association was prominent in posterior brain regions but not in the frontal lobes [20]. This latter study differed from ours in the method for measuring processing speed: Whereas we used spatial judgment tasks that did not rely upon memory, they used a computerized N-back test that relied on working memory, as well as a non-memory, non-computerized letter or pattern matching task. While we do not know the reason why their findings diverged from ours, we speculate that the inclusion of a memory-dependent test sampled more than simple cognitive processing speed. Early, preclinical Alzheimer’s disease, which is inevitably present in any large sample of older subjects, is an example of a process that could result in secondary axonal damage and poor memory.

Another prior report emphasized the importance of white matter lesion volume and white matter atrophy in causing apparent changes in FA with age [48]. However, we observed no correlation between these factors and cognitive processing speed (Figure 6B, C), perhaps because of the very minimal extent of visible white matter lesions among our subjects. Others have suggested that in the process of age-related white matter deterioration, decreases in FA may precede and be more sensitive than volume loss [6], [11], and so it is possible that subjects with lower FA values and normal-appearing white matter will go on to accumulate visible white matter lesions, a consideration that should factor into any longitudinal follow-up. We measured white matter lesion volume using a T1 sequence, which is less sensitive than FLAIR; we may therefore have underestimated the extent of subcortical ischemic disease. On the other hand, while we did not have FLAIR images on all of our participants, we did have this sequence on the individual who, of all our participants, had the greatest white matter lesion load; that FLAIR image is illustrated in Figure 6C and reveals only mild disease.

By including age as a confound regressor in voxel-wise regressions of FA with cognitive processing speed (Figure 3), we show an age-independent effect of white matter integrity (see also [19]). Therefore, other factors must contribute to differences in white matter integrity among healthy, older adults. It is likely that certain environmental factors, such as vascular risk factors, may contribute. Also likely – but currently unexplored – is a role for genetic factors, including polymorphisms in genes that affect central myelination.

Our study included some limitations: First, the number of subjects was not uniformly distributed across the age spectrum; many subjects were clumped in the late 60s, resulting in less information about individuals at the upper and lower ends of the age spectrum under consideration. Second, we used a very narrow definition of “normal” in order to achieve as homogeneous a sample population as possible, and our findings may not necessarily be applicable to more general populations of older adults. Third, despite the methodological advantages of voxel-wise cross-subject statistics afforded by TBSS, some information is inevitably lost during image coregistration. Future work may address these limitations, and also identify the factors responsible for age-related white matter changes. Hopefully these future insights will lead to interventions to stem the slowing of cognitive processing and its associated morbidity.
